# Hypothesis and theory: mechanical instabilities and non-uniformities in hereditary sarcomere myopathies

**DOI:** 10.3389/fphys.2014.00350

**Published:** 2014-09-15

**Authors:** Alf Månsson

**Affiliations:** Department of Chemistry and Biomedical Sciences, Linnaeus UniversityKalmar, Sweden

**Keywords:** myopathy, striated muscle, force-velocity relationship, actomyosin, heart, skeletal muscle

## Abstract

Familial hypertrophic cardiomyopathy (HCM), due to point mutations in genes for sarcomere proteins such as myosin, occurs in 1/500 people and is the most common cause of sudden death in young individuals. Similar mutations in skeletal muscle, e.g., in the *MYH7* gene for slow myosin found in both the cardiac ventricle and slow skeletal muscle, may also cause severe disease but the severity and the morphological changes are often different. In HCM, the modified protein function leads, over years to decades, to secondary remodeling with substantial morphological changes, such as hypertrophy, myofibrillar disarray, and extensive fibrosis associated with severe functional deterioration. Despite intense studies, it is unclear how the moderate mutation-induced changes in protein function cause the long-term effects. In hypertrophy of the heart due to pressure overload (e.g., hypertension), mechanical stress in the myocyte is believed to be major initiating stimulus for activation of relevant cell signaling cascades. Here it is considered how expression of mutated proteins, such as myosin or regulatory proteins, could have similar consequences through one or both of the following mechanisms: (1) contractile instabilities within each sarcomere (with more than one stable velocity for a given load), (2) different tension generating capacities of cells in series. These mechanisms would have the potential to cause increased tension and/or stretch of certain cells during parts of the cardiac cycle. Modeling studies are used to illustrate these ideas and experimental tests are proposed. The applicability of similar ideas to skeletal muscle is also postulated, and differences between heart and skeletal muscle are discussed.

## Introduction

**Figure 1:**
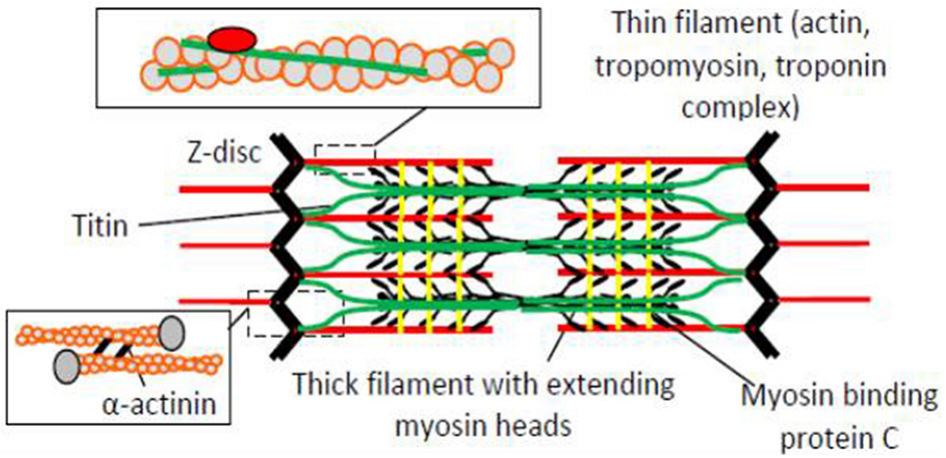
**The sarcomere, the highly ordered functional unit of striated muscle with key protein components schematically illustrated**. The resting length of the sarcomere is approximately 2.0 μm in the human heart and 2.5 μm in human skeletal muscle.

Hereditary heart diseases, cardiomyopathies, with mutations in genes for key proteins in the muscle sarcomere (Figure 1) occur in as many as 1/500 people and are the most common cause of sudden death in young individuals (Harvey and Leinwand, 2011; Teekakirikul et al., 2012). Some of the most commonly affected proteins include ventricular β-myosin heavy chains, myosin binding protein C, troponin I, troponin T, tropomyosin, and myosin regulatory light chains (Xu et al., 2010). While much attention has been directed to sarcomere myopathies in the heart, the insight into their genetic basis (Geisterfer-Lowrance et al., 1990) triggered interest into related skeletal muscle diseases (Cuda et al., 1993; Martinsson et al., 2000). The consequences of these skeletal muscle sarcomere myopathies may be equally severe with, grave disability, respiratory failure and death at a young age (Laing and Nowak, 2005b; Laing, 2007; Ochala, 2008; Tajsharghi and Oldfors, 2013). Interestingly, however, a large majority of the myosin mutations that cause severe cardiomyopathy, only cause mild skeletal muscle disease (Oldfors, 2007). Severe disease in skeletal muscle is generally associated with mutations in thin filament proteins such as nebulin (not present in heart), actin, or the regulatory troponin-tropomyosin complex (Ochala, 2008).

Importantly, both in heart and skeletal muscle, severe disease often does not develop until adolescence or adulthood (Frey et al., 2012). This offers a time window that should be useful for therapeutic interventions. However, such options are currently hampered by lack of insight into the mechanisms whereby the minor, primary disturbances in the sarcomere proteins are transformed into disease with appreciable morphological changes (Laing and Nowak, 2005a; Ochala, 2008; Frey et al., 2012; Teekakirikul et al., 2012). In the heart there is, for instance, severe hypertrophy with increased wall thickness and cell volume (hypertrophic cardiomyopathy-HCM) or dilation with elongated cells and larger ventricular cavities (dilated cardiomyopathy-DCM). The changes in HCM are associated with myofibrillar disarray and fibrosis throughout the myocardium (Ashrafian et al., 2011; Frey et al., 2012; Teekakirikul et al., 2012) but also with microvascular disease and defects in the mitral valves (Ashrafian et al., 2011). In skeletal muscle, the picture in myopathies is diverse both clinically and morphologically (Laing and Nowak, 2005a; Oldfors, 2007; Ochala, 2008; Marston et al., 2013; Tajsharghi and Oldfors, 2013). Here, the focus will be primarily on hereditary HCM due to appreciably higher prevalence than hereditary DCM. The developed ideas will also be considered in relation to hereditary skeletal muscle diseases with mutations in sarcomeric proteins. It is important to note that the term “HCM” will only refer to hereditary disease. It is not used to denote acquired diseases associated with cardiac hypertrophy, for example in pressure overload.

One major reason why the mechanisms underlying HCM and skeletal muscle myopathies remain elusive is the appreciable multidimensional complexity in spite of a simple primary defect in the form of a point mutation. First of all, the time dependent secondary changes most likely start already during embryonic development (Olivotto et al., 2009). These changes continue throughout life, while being modulated by a range of environmental, genetic and epigenetic factors that may modify disease penetrance and where each is likely to vary with time (Cooper et al., 2013). This time dependence and variability in clinical course has contributed to the difficulty of obtaining a clear and comprehensive picture of the pathogenesis even for a given point mutation. The picture is further blurred by the additional dimension that the path from a given isolated mutation to full-blown disease seems to vary between humans and typical experimental animals, e.g., transgenic mice (Lowey et al., 2008, 2013; Spudich, 2014). Adding further to the complexity, recent studies have also suggested that the proportional expression of wild-type and mutated protein may vary between cells in the same individual (Kirschner et al., 2005), and that there is allelic imbalances with different proportional expression of the mutated protein in different heterozygous individuals (Casadonte et al., 2006; Tripathi et al., 2011; Di Domenico et al., 2012). Considering all the above, it is not surprising that it is highly challenging to arrive at a clear understanding of the pathogenesis of the sarcomere myopathies. Indeed, there are even conflicting views on how a given myosin mutation affects the actomyosin cross-bridge function and whether it leads to gain or loss of function (Wang et al., 2003; Lowey et al., 2008, 2013; Sommese et al., 2013b) (reviewed by Spudich, 2014). Part of the reason for the discrepancies seems to be that a given myosin mutation has different effects depending on the myosin isoform background, a property that varies between species due to differences in dominant ventricular isoform (Lowey et al., 2008, 2013). Recent insights gained using expression of wild-type and mutated proteins from both humans and key experimental animals seem to suggest that there is a tendency (Kohler et al., 2003; Marston, 2011; Teekakirikul et al., 2012; Bai et al., 2013; Spudich, 2014), although with exceptions (Greenberg et al., 2010), for HCM mutations in myosin and regulatory proteins to cause increased rather than reduced power output and/or increased rather than reduced Ca-sensitivity. There is also some evidence for early hypercontractility on the whole organ level (Teekakirikul et al., 2012) although the evidence is not unequivocal (Witjas-Paalberends et al., 2013). The association between increased diastolic pressure (related to, so called, diastolic heart failure) in HCM and an increased Ca-sensitivity (Ferrantini et al., 2009; Ashrafian et al., 2011; Harvey and Leinwand, 2011; Marston, 2011; Frey et al., 2012; Lovelock et al., 2012; Teekakirikul et al., 2012; Bai et al., 2013; Memo and Marston, 2013; Witjas-Paalberends et al., 2013; Sommese et al., 2013a; Spudich, 2014) seems to have gained more general, although not universal (Kirschner et al., 2005), acceptance. However, in this connection it should be mentioned that diastolic dysfunction can also be caused by changes in titin function. This has been demonstrated in mouse models where shortening of the titin spring region (Chung et al., 2013) caused increased ventricular wall stiffness. Moreover, in clinical studies (Borbely et al., 2009) hypophosphorylation of a specific titin isoform increased resting tension with possible implications for diastolic dysfunction in cardiomyocytes from failing human myocardioum.

A majority of the secondary remodeling effects in hereditary HCM are reminiscent of those in pressure overload such as caused by hypertension and aortic stenosis. This includes hypertrophy and fibrosis as well as changes in gene expression profile to a more fetal pattern (cf. Bernardo et al., 2010; Harvey and Leinwand, 2011). A difference is the often more patchy and regional appearance of the secondary changes in HCM due to sarcomeric protein mutation. In cardiac hypertrophy due to pressure overload, mechanical stimuli affecting the myocytes are believed to be major initiators of changes in cell signaling that lead to hypertrophy (Bernardo et al., 2010).

An important question that remains to be answered is why similar signaling pathways as in pressure overload would be activated in HCM (Ferrantini et al., 2009; Olivotto et al., 2009; Ashrafian et al., 2011; Harvey and Leinwand, 2011; Frey et al., 2012; Moore et al., 2012; Teekakirikul et al., 2012). Here, I hypothesize that the initiating stimuli in HCM are similar as in pressure overload but more localized, i.e., they involve increased mechanical stress that only affects a limited fraction of the cardiomyocytes. I further use model studies for initial tests as to whether this hypothesis is consistent with either or both of the following ideas: (i) that altered kinetics of the actomyosin interaction causes critical instabilities in the force-velocity (F-V) relation at the sarcomere level (Julicher and Prost, 1995; Vilfan et al., 1999; Mansson, 2010) and/or (ii) that certain cells in the heart or segments of skeletal muscle fibers are stretched during overall isometric/isovolumetric contraction due to non-uniformity in force-generating capacity between cells (Kirschner et al., 2005).

## Cell signaling and phenotype in pressure overload and in HCM—similarities and differences

Stimulation of angiotensin II (AT-II) receptors appears to be central in the signaling pathways that lead to hypertrophy, fibrosis etc. in HCM, as well as in pressure overload and, at least in, pressure overload, it seems clear that this signaling is initiated by mechanical stress (Lijnen and Petrov, 1999; Lim et al., 2001; Amedeo Modesti et al., 2002; Zou et al., 2004; Bernardo et al., 2010; Ashrafian et al., 2011; Harvey and Leinwand, 2011; Weeks and McMullen, 2011; Teekakirikul et al., 2012; Luo et al., 2013). The down-stream effects are enhanced or complemented by the action of noradrenaline and endothelin on other G-protein coupled receptors and possibly by paracrine substances released from non-cardiomyocyte cells (Teekakirikul et al., 2012; Cilvik et al., 2013). These effects involve calcium dependent calcium/calmodulin kinase and calcineurin as well as a range of transcription factors ultimately leading to marked changes in protein expression (Bernardo et al., 2010; Harvey and Leinwand, 2011). Importantly, these pathologic pathways largely differ from those in physiologic hypertrophy (“athletes heart”) which seem to mainly involve activation of the Akt-mTOR pathway (Bernardo et al., 2010; Weeks and McMullen, 2011).

The similarities in phenotype (hypertrophy associated with fibrosis and similar changes in protein expression pattern) between HCM and pressure overload as well as the involvement of similar signaling systems in the two cases seems undisputed on the basis of a survey of the literature. However, whereas the mechanical stimulus that initiates such signaling seems quite straightforward in pressure overload the initiating mechanism is less evident in HCM. It has been suggested that reduced energy efficiency of contraction is of importance in this regard but it is not clear how (Jung et al., 1998; Sweeney et al., 1998; Crilley et al., 2003; Frey et al., 2006; Luedde et al., 2009; Harvey and Leinwand, 2011; Marston, 2011; Frey et al., 2012; Teekakirikul et al., 2012). Another possibility that has also been widely considered is that disturbances in Ca-homeostasis, as a result of the mutations, could be a triggering factor (Ferrantini et al., 2009; Ashrafian et al., 2011; Harvey and Leinwand, 2011; Marston, 2011; Frey et al., 2012; Teekakirikul et al., 2012). The latter disturbances may be secondary to inefficient energy usage which may reduce calcium pumping by the sarcoplasmic reticulum ATPase. They may also be understood more directly for mutations that alter the Ca-affinity of troponin (Marston, 2011) thereby not only affecting the Ca-sensitivity of contraction but also changing the calcium buffering capacity.

## Statement of hypothesis

I propose here an alternative hypothesis with the aims of supplementing existing ideas (cf. above) and stimulating new experiments and modeling studies. To the best of my knowledge this hypothesis has not been considered previously, at least not explicitly, but it has been stimulated by recent findings (Kirschner et al., 2005; Mansson, 2010) (see below for details).

I hypothesize that in HCM: (1) similar signaling pathways (associated with AT-II) operate as in pressure overload, but less homogeneously over the ventricular wall and (2) that the dominant initiating stimulus that activates these pathways is similar to that in pressure overload, i.e., increased cellular stress/stretch. The major distinguishing feature, compared to pressure overload, is the idea that the mechanical effects act locally on sub-populations of the cells, rather than globally on all ventricular cells.

## Initiation of pathologic signal transduction in HCM

**Figure 2:**
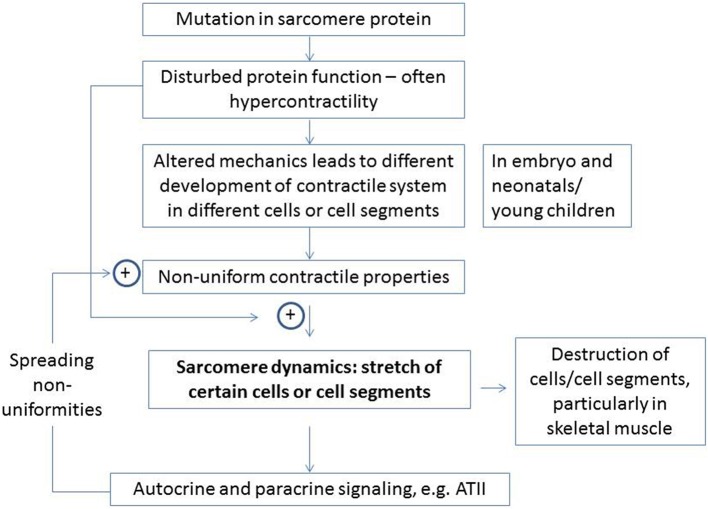
**Major elements of proposed hypothesis for development of HCM and related diseases in skeletal muscle**. The enhancement of certain effects by specific elements is indicated by plus signs. Details are considered in the text.

Two mechanisms are considered (see sub-sections below) that may mediate mechanical initiation of pathologic signal transduction. The major components of the overall hypothesis and the two mechanisms are summarized in Figure 2. The possible relation to skeletal muscle myopathies is discussed in a separate section at the end of the paper but is also considered briefly in other sections.

### Instability on sarcomere level

**Figure 3:**
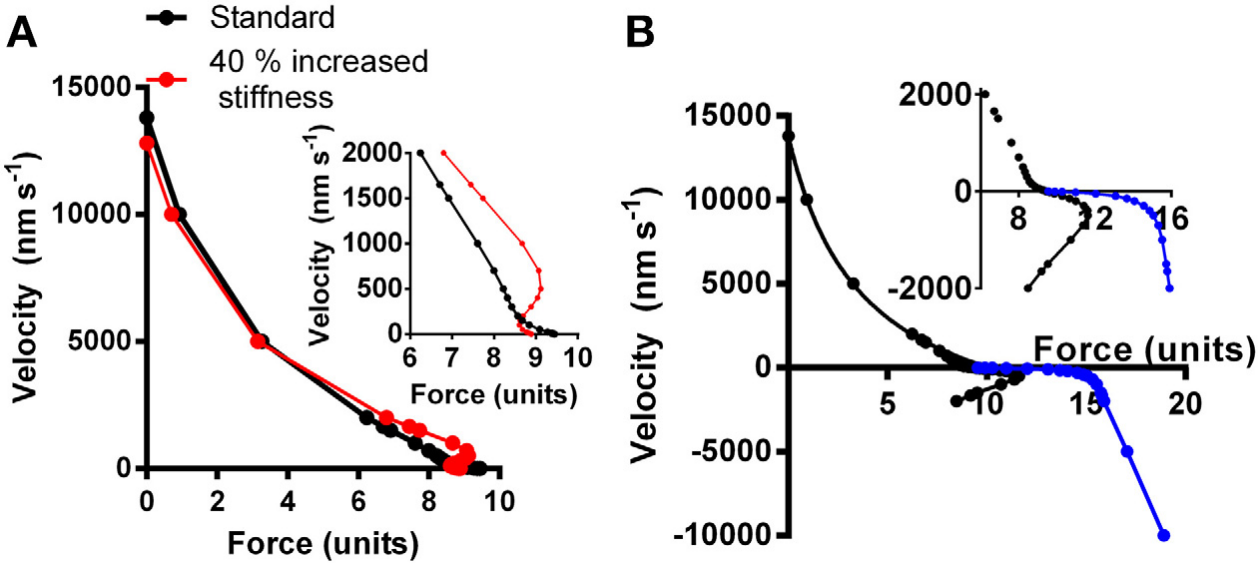
**Simulated relationship between force and shortening (positive) and lengthening velocity (negative). (A)** Black symbols and connecting lines: standard parameter values (Table S1; corresponding to normal healthy fast skeletal muscle). Red symbols and connecting lines: standard parameter values but cross-bridge stiffness increased by 40% without further changes in parameter values. Inset: simulated force-velocity data in the high force region shown in greater detail. **(B)** The force-velocity relationship for the standard conditions in **(A)** re-plotted together with simulated lengthening part of the relationship either assuming same attachment rate as during shortening and isometric contraction (Equation S4a; black symbols) or assuming increased attachment rate with increased velocity of lengthening (Equation S4b; blue symbols). The latter data are reasonably consistent with experimental data (Lombardi and Piazzesi, 1990) whereas the former are not.

Model studies (Julicher and Prost, 1995; Vilfan and Frey, 2005; Mansson, 2010) have suggested that certain combinations of parameter values that govern the actomyosin interactions may produce mechanical instabilities. These are characterized by anomalous load-velocity (F-V) relationships of the muscle with more than one stable velocity for a given load. This is illustrated in Figure 3 for a model similar to that in (Mansson, 2010). Here, the black curve simulates a “normal” F-V relationship based on the parameter values in Table S1 whereas the red curve simulates a relationship with instability at loads close to the isometric (see below and legend of Figure 3 for details).

As pointed out previously (Mansson, 2010), regions of positive or infinite derivative of velocity with respect to force dV/dF (Figure 3) may be deleterious for normal muscle function with unpredictable effects and potential difficulties of regulation. The effect may be critical if sarcomeric units in series have different force-generating capacity. This is observed physiologically (see below) and it is proposed here, that the phenomenon is enhanced in myopathies.

The normally low magnitude of the negative slope |dV/dF| of the F-V relationship for lengthening and in the high-force region for shortening is probably important in order to reduce sarcomere-length redistribution during contractions where isometric or almost isometric tension is developed (Edman et al., 1997). This stabilizing effect follows from appreciable drop in tension-generating capability with a small increase in shortening velocity of the strongest fiber segments (the ones that shorten) and a high increase in resistance to stretch of elongating segments. Without such an inherent stabilization, the weakest segments may become severely overstretched with potential damage to the cell or strong local stimulation of hypertrophic signaling. The latter would be expected because stretch of a weak cell during activity will cause stretch of titin in this cell as well as high tension in the individual filaments and Z-lines, structures known to be associated with tension-, and strain-sensing (Luther, 2009; Linke and Kruger, 2010; Ottenheijm et al., 2011; Raskin et al., 2012).

A recently reported loss of the Frank-Starling mechanism in HCM (Sequeira et al., 2013) may also contribute to sarcomere instability similar to that seen with an anomalous F-V relationship. Thus, in normal cardiac muscle, the Frank-Starling mechanism immediately (Mateja and De Tombe, 2012) increases Ca-sensitivity of contraction upon increased sarcomere length, i.e., the strength of any weak segments that undergo stretch is increased, counteracting excessive sarcomere redistributions. It was suggested that the lost capacity to respond to increased filling pressure in HCM in the case of a blunted Frank-Starling mechanism might, in itself, initiate compensatory hypertrophy (Sequeira et al., 2013). On the other hand, the phenomenon would clearly also contribute to the mechanism proposed here and it may be important in pathogenesis if the Frank-Starling mechanism is blunted not only in HCM patients but also in healthy mutation carriers. As mentioned above, a range of HCM mutations cause rather subtle changes in the kinetics and mechanical properties of the actomyosin cross-bridges. In order to investigate whether such effects may cause instabilities in the F-V relationship, as described above, we performed a number of simulations using a model developed from recent work (Albet-Torres et al., 2009; Mansson, 2010; Persson et al., 2013) (Supporting Information). First, we found that isolated changes in only a limited number of the parameter values (Tables S1, S2) could reproduce instabilities in the F-V curve of the type illustrated in Figure 3, with only small simultaneous changes in maximal tension and maximal velocity, typically seen in HCM. Of particular interest in this connection was an isolated increase (~40%) in cross-bridge stiffness that causes an anomalous F-V relationship (red, Figure 3A) with regions of positive slope and the mentioned type of instability with more than one stable velocity for a given load. This effect is due to the fact that the cross-bridge, after attachment, has to overcome an increased free-energy barrier (Figure S1), as result of increased stiffness, in stretching the elastic element before entering into the main force-generating state. This energy barrier is lowered by the reduced cross-bridge strain during shortening which explains why force can be as high or higher during slow shortening (red, Figure 3A) than in isometric contraction.

### Non-uniformity between cardiac muscle cells or along skeletal muscle fibers

Non-uniformity in strength of contraction between different cardiac muscle cells in series or along a skeletal muscle fiber, for whatever reason, will inevitably lead to shortening and elongation of the strongest and weakest segments, respectively if the muscle as a whole undergoes isometric contraction. The phenomenon exists physiologically along skeletal muscle fibers where it has been studied in appreciable detail (Gordon et al., 1966; Julian and Morgan, 1979; Edman and Reggiani, 1984; Edman et al., 1985, 1988). It has also been studied in mammalian myofibrils from heart and skeletal muscle (Stehle et al., 2002; Poggesi et al., 2005). Furthermore, two populations of cells differing with respect to certain contractile properties have been observed among porcine cardiomyocytes (McDonald et al., 2012).

The non-uniformity in contractile properties between different cardiac myocytes and along a skeletal muscle fiber has important physiological roles. This includes effects to speed up relaxation after an isometric contraction (Edman and Flitney, 1982; Poggesi et al., 2005) and to contribute to some aspects of the resistance to stretch (Campbell et al., 2011; Edman, 2012; Rassier, 2012). However, excessive stretch of some muscle segments associated with non-uniformities may also be the basis of cellular damage (Macpherson et al., 1997).

Interestingly, increased variability in calcium sensitivity (reported as pCa) was observed (Kirschner et al., 2005) between different skeletal muscle fibers of a given patient either affected by an Arg723Gly, Arg719Trp, or an Ile736Thr mutation in the β-cardiac/slow myosin gene (MYH7). In the discussion they (Kirschner et al., 2005) stated: “the variability in pCa(50) from fiber to fiber is likely to cause imbalances in force generation and be the primary cause for contractile dysfunction and development of disarray in the myocardium.”

One factor contributing to non-uniform contractile properties between different muscle segments under physiological conditions is differential expression of different myosin isoforms. For instance, different myosin isoform composition has been observed along the length of individual skeletal muscle fibers associated with the protein expression control by different nuclei of these multinuclear cells (Edman et al., 1988; Rosser and Bandman, 2003).

Related to this observation, Kirschner et al. (2005) also considered whether the non-uniformities in pCa between cells may be attributed to different content of mutated protein. Such effects may occur in heterozygous individuals with HCM if there is different fractional expression of mutated and wild-type protein in different cardiac cells or along a given skeletal muscle fiber (as used by Kirschner et al., 2005). However, it is not straightforward to reconcile the idea with findings (Fatkin et al., 1999; Ho et al., 2000; Richard et al., 2000; Nanni et al., 2003) that patients and experimental animals, who are homozygous for a given mutation, exhibit a more severe phenotype than those who are heterozygous. This finding suggests that the amount of mutated protein *per se*, is the critical factor. This idea is also consistent with positive correlation (Casadonte et al., 2006; Tripathi et al., 2011; Di Domenico et al., 2012) between the phenotype severity and the fractional expression of mutated protein in heterozygous individuals, although other interpretations (Tripathi et al., 2011) are possible. The increased severity with increased fraction of mutated protein also seems to accord with recent findings that RNA interference based inhibition of the expression of mutated myosin prevents development of HCM upon just 25% reduction in the level of the mutant transcripts (Jiang et al., 2013).

Of course, in spite of the above findings, one cannot exclude the possibility that non-uniform expression of mutated and wild-type myosin in different cells may affect disease severity in heterozygous individuals or that the pathogenic mechanisms differ between the homozygous and heterozygous cases. However, if it will be possible to conclusively demonstrate that the effect of non-uniform expression is negligible and that it is the overall amount of mutated protein that is important, the findings of Kirschner et al. (Kirschner et al., 2005) of inter-cellular variability must be explained by another mechanism. One may here consider overall changes in cytoarchitecture that give variations in force between cells or between cell segments, consistent with a developmental origin (Olivotto et al., 2009). Another, basis for the effect may be related to the myofibrillar disarray observed upon incorporation of myosin with HCM mutations into embryonic chicken myocytes (Wang et al., 2003), or upon drug induced (instead of mutation induced) modifications of contraction kinetics of embryonic myocytes (Rodriguez et al., 2013). Similar myofibrillar disarray in human cardiomyocytes during embryonic development in the presence of HCM mutations may account for the variability if different cells or cell segments are affected by disarray to different degree. Irrespective of the reason for variability between cells, the weakest cells or cell segments will be stretched by the remaining cells during isovolumetric/isometric contraction, an effect that might be worsened by changes to the load-velocity relationship as indicated in Figure 3. Such stretch would be sensed by tension-, and strain-sensing systems in the sarcomeres (Luther, 2009; Linke and Kruger, 2010; Ottenheijm et al., 2011) (see above) and elsewhere, e.g., in the cell membrane (Bernardo et al., 2010) with initiation of hypertrophic signaling. Areas with mechanical non-uniformity and local activation of such signaling pathways may then function as foci from which areas of hypertrophy, fibrosis and disarray on the myocyte level spread (Figure 2). This course of events is consistent with focal areas of abnormal strain and fibrosis seen within the limited resolution of cardiac imaging in patients with fully developed HCM (Nagakura et al., 2007; Ghio et al., 2009; Maron et al., 2009). Of even greater interest, regional and patchy irregularities in morphology (Germans et al., 2006) (without hypertrophy) and ventricular wall strain during both systole and diastole have also been observed (Cardim et al., 2002; Yiu et al., 2012; Forsey et al., 2013) in mutation carriers. These changes have particularly been seen in those regions of the ventricle where hypertrophy and fibrosis is most severe late in the course of the disease.

A simplified model (Supplementary Material) was used in order to elucidate the effect of non-uniform contractile properties between cardiac myocytes (~100 μm long) in series or along several cm long skeletal muscle fibers. Here we approximated this complex arrangement by assuming that two sarcomeres with different contractile properties act in series.

**Figure 4:**
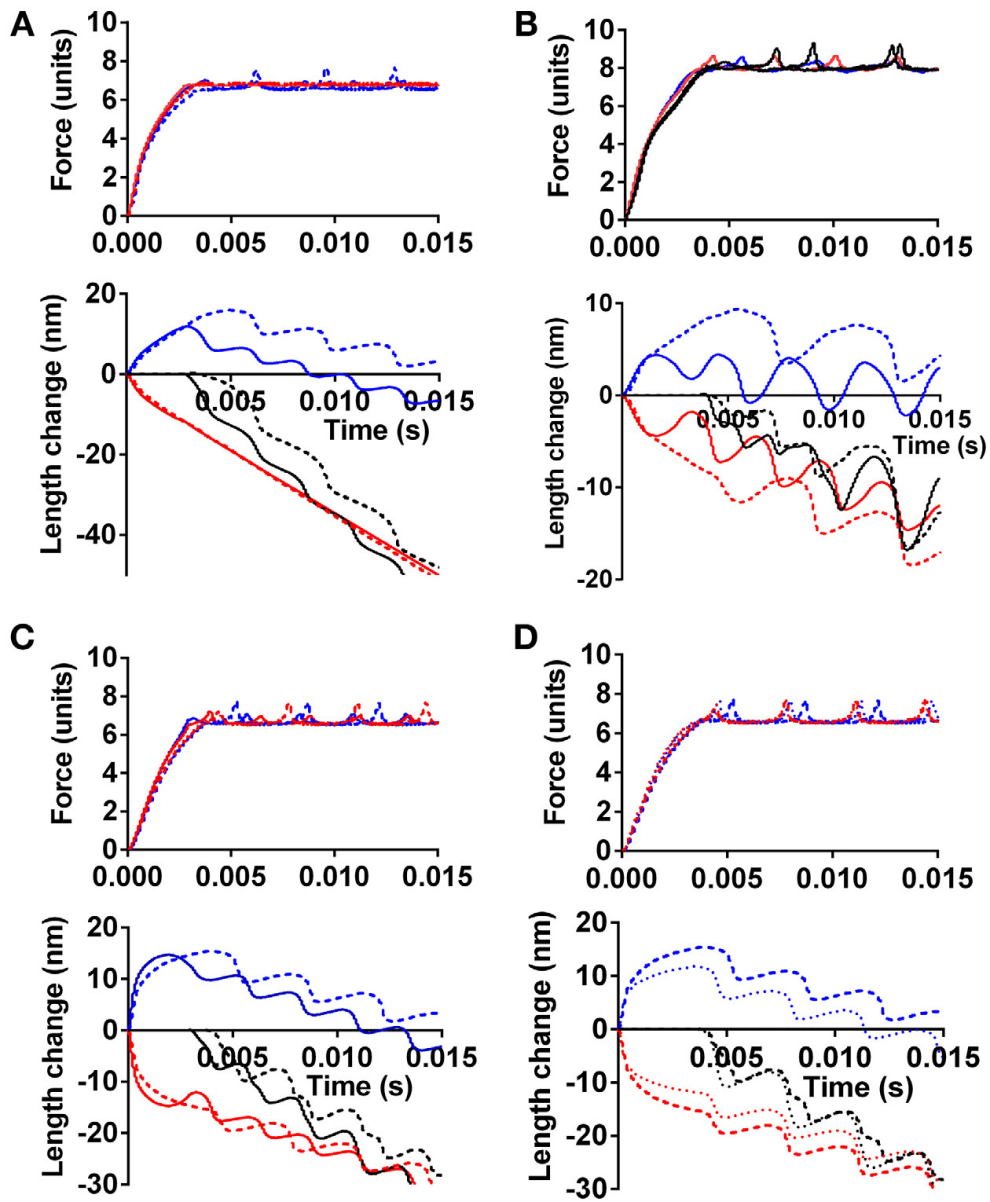
**The effect of different force-generating capacity or calcium sensitivity of two cells in series on simulated tension and length response. (A)** Effect of 30% higher maximum tension of one cell. Force clamped to 70% of maximum isometric force of weaker cell. Full lines: Force-velocity curve as black symbols in Figure 3. Dashed lines: Force-velocity curve as red symbols in Figure 3. Blue: response of cell with lowest tension-generating capacity. Red: response of strongest cell. Black in length-time plot: sum of length change for both cells in series. **(B)** Similar to **(A)**, but only 10% higher maximum tension of one cell and force clamped to 85% of maximum isometric force of weaker cell. Line formats and color codes as in **(A)**. Full calcium activation assumed from onset of simulation. **(C)** Simulated time course including activation process with higher calcium sensitivity of contractile system in one cell (red) but similar maximum isometric tension in both cells. Line formats as in other panels. **(D)** Dashed lines, same as dashed lines in **(C)**. Dotted lines, same as in **(C)** but velocity-dependent increase in attachment rate during stretch as described in text.

Figure 4 illustrates the effect of different force-generating capacity of the two sarcomeres (corresponding to cells or cell-segments) in series and the effect under these conditions of instability in the F-V relationship (red symbols in Figure 3). In Figure 4A, the tension and length changes of the two cells were simulated assuming that one of the sarcomeres develops 30% higher maximum force than the other, e.g., corresponding to more myofibrils in parallel or more correctly oriented myofibrils on the cellular level. The results are shown for both cases with a stable (black line in Figure 3) and instable (red line in Figure 3) F-V relationship of both sarcomeres. In Figure 4B similar results are shown as in Figure 4A but with only 10% higher force development of one sarcomere compared to the other, and with clamping of the force to 85% (instead of 70% in Figure 4A) of the maximum isometric force of the weakest sarcomere. It can be seen in both Figures 4A,B that the stronger sarcomere shortens, stretching the sarcomere in series during the overall isometric phase of contraction. It can also be seen that this effect is enhanced with the anomalous F-V curve (red curve in Figure 3), particularly when the load is clamped to the higher level and when the difference in maximum force-generating capacity is small (Figure 4B).

Effects of calcium-activation are included in the simulations in Figure 4C, for cases with a stable (black line in Figure 3) and instable (red line in Figure 3) F-V relationship. As expected, the sarcomere with highest calcium-sensitivity shortens during the isometric phase (corresponding to isovolumetric phase in the heart), stretching the sarcomere in series, in spite of the same maximum isometric tension in the two sarcomeres. Here, the instability of the F-V relationship only had a small enhancing effect on the amount of stretch of the weak sarcomere during the overall isometric phase of the contraction. This may be due to reduced instability at low level of activation, due to assumed calcium dependence of the attachment rate constant in these simulations. Importantly, not all models of activation include this assumption (Cecchi et al., 1981).

In the simulations above, it was assumed that the rate of myosin head attachment was not increased by stretch. If, instead, the attachment rate was assumed to increase with lengthening velocity, a type of effect found necessary in previous models (Lombardi and Piazzesi, 1990; Mansson, 2010) (see also Figure 3), the sarcomere length redistribution was reduced in amplitude as illustrated in Figure 4D.

## Implications, limitations, and potentials of model studies

The present model-simulations merely aim to illustrate the type of effects that may be seen due to sarcomere instabilities and non-uniform contractile properties between myocardial cells, or along a skeletal muscle fiber rather than simulating the exact behavior of human muscle. This limitation in scope is largely due to the limited availability of experimental data for full definition of the models under physiological conditions. This particularly applies to human cardiac muscle but also to an appreciable extent to mammalians skeletal muscle (Supporting Information). Thus, whereas model studies at present have certain limitations, on-going developments are likely to increase their usefulness. In particular, kinetic and elastic properties of both wild-type and mutated cardiac myosins are in the process of being elucidated in similar detail to fast skeletal muscle myosin (Deacon et al., 2012; Bloemink et al., 2013; Sommese et al., 2013b). Similar developments are underway (Bai et al., 2013) for the contractile effects of e.g., regulatory thin filament proteins.

Clearly, to refine the model in order to critically evaluate the current idea that some sarcomeres are stretched by others, it will be essential to increase of the understanding of the lengthening part of the load-velocity relationship and establish whether cardiomyopathy mutations might affect this part (cf. Figures 4C,D).

Better models based on more complete experimental data should give further insights into the effect of the myopathy mutations in otherwise normal cells, before they are affected by remodeling. The models would allow extrapolation from single molecule results to ensemble function and also allow the incorporation of effects on higher level of hierarchial organization than that of sarcomeres and half-sarcomeres.

## Suggested experimental tests

In studies of cardiomyopathies, using isolated proteins, limited effort has, so far, been devoted to the detailed shape of the load-velocity relationship for shortening as well as lengthening. Studies of F-V data have mainly utilized semi-quantitative methods with so called loaded motility assays, where actin-binding proteins impose a frictional load between the surface and actin filaments (Bing et al., 2000; Sommese et al., 2013b). Whereas this technique detects gross changes in maximum power output it seems less likely that it will detect more subtle changes in shape of the force velocity relationship such as exemplified in Figure 3. In order to detect the types of instabilities considered here, high-resolution studies will be required to obtain F-V data from small ensembles of contractile proteins. The use of small ensembles is important because the F-V relationship is a property of an ensemble of actomyosin motors (Huxley, 1957; Eisenberg and Hill, 1978; Vilfan et al., 1999).

An interesting question, in terms of non-uniformities between cardiomyocuytes, is whether there is allelic expression of myosin so that some cells express the wild type and some the mutant or whether there is variable expression of both within one cell (Kirschner et al., 2005). However, whatever the cause of non-uniformities between cells, it is clear from the simulations that they would lead to increased lengthening of some cells and shortening of others during overall isovolumetric/isometric contraction. One would thus expect increased non-uniformity in strain during systole in the whole heart of healthy human mutation carriers, (cf. Ghio et al., 2009). Further insight in this regard relies on further improvements of methods for cardiac imaging. Increased non-uniformity of sarcomere behavior during contraction should also be possible to detect in cardiac ventricular strips from experimental animals and between segments along a skeletal muscle fiber. Another interesting test would be if a given degree of expression of a mutated myosin in e.g., a transfected embryonic cardiomyocyte (Wang et al., 2003) leads to increased variability in the degree of myofibrillar disarray indicating the possibility of non-uniformities developing embryologically.

One may also consider attempts to probe how activation of signal transduction systems is spatially and temporally distributed over the cardiac ventricle in experimental animals with HCM causing mutations. Finally, it may be of interest to consider the option to control the process in greater detail by actually inducing local non-uniformities in contractile function *in situ*, perhaps by localized adeno-associated virus (AAV) based transfection of relevant genes. The animals should then be followed up with regard to the presence of non-uniform contraction, localized AT-II signaling and, finally, development of a HCM phenotype.

## Skeletal muscle myopathies

Skeletal muscle sarcomere myopathies are due to mutations in similar genes as cardiomyopathies but the mutations that predominantly cause severe disease differ. Whereas myosin and myosin-binding protein C are mainly affected in HCM (see above) it is mainly thin filament proteins such as nebulin (not present in heart), actin, tropomyosin, troponin T and troponin I that are affected in severe sarcomere myopathies in skeletal muscle (Laing, 2007; Ochala, 2008). It is particularly puzzling that that even malignant HCM-mutations e.g., in the MYH7 gene, with modified ventricular β-myosin heavy chain, identical to the slow skeletal muscle myosin heavy chain, often give a mild skeletal muscle phenotype (Oldfors, 2007). One may speculate about reasons for this, from differences in signaling cascades that are activated by the mutation over different loading conditions of the different muscles to different compensatory mechanisms, i.e., involving altered state of phosphorylation of a range of regulatory proteins in the heart and compensatory use of non-slow motor units in skeletal muscle. These are interesting questions that may teach us lessons about the pathogenesis but they are not a major focus here.

The most common among the rare skeletal muscle sarcomere myopathies with bewildering morphological and clinical consequences are denoted nemaline myopathy and distal arthrogryposis. These are caused by a range of mutations in the thin filament proteins. The nemaline myopathies, in addition to being characterized by specific morphological changes late in the course of the disease, with so called nemaline bodies (Ochala, 2008), containing actin and sarcomere Z-line proteins seem to exhibit reduced Ca-sensitivity and hypocontractility (Ochala, 2008; Ochala et al., 2012; Memo and Marston, 2013). In contrast, distal arthrogryoposis is characterized by increased Ca-sensitivity and hypercontractility (Robinson et al., 2007; Ochala, 2008; Memo and Marston, 2013), e.g., with contractures in distal muscles. For other skeletal muscle diseases, the reader is referred to a number of comprehensive recent reviews (Laing and Nowak, 2005a; Laing, 2007; Oldfors, 2007; Ochala, 2008; Wallgren-Pettersson et al., 2011; Tajsharghi and Oldfors, 2013).

In spite of the differences from cardiomyopathies, I postulate that the basic mechanisms for muscle destruction in several skeletal muscle diseases, caused by mutations in sarcomere proteins, are similar to the hypertrophy response in heart, i.e., instabilities on the sarcomere level and non-uniformities along the length of the muscle. This is consistent with the fact that the results of Kirschner et al. (2005), on basis of which they proposed that non-uniformities are important in cardiomyopathies, were actually derived using slow skeletal muscle preparations.

If there are increased differences in force-generating capability along skeletal muscle fibers with sarcomere myopathy mutations one would expect increased sarcomere re-distributions within the fiber with the risk that some segments are overstretched (see above for mechanisms). In skeletal muscle fibers it is also known that stretch could stimulate hypertrophy (Roig et al., 2009). On the other hand, extensive non-uniform stretch, possibly facilitated by instabilities of the F-V relationship may lead to cell damage. Indeed, cell damage, rather than hypertrophy may be even more likely in skeletal muscle than in the heart as the Frank-Starling mechanism, giving increased Ca-sensitivity with stretch, may counteract the stretch in heart muscle but is not present in skeletal muscle.

## Conclusions

The present paper has analyzed a hypothesis according to which the early compensatory changes in HCM occur due to local stimulation of cell-signaling mechanisms similar to those in pressure-overload. According to this hypothesis, locally increased mechanical stress on the cardiomyocytes is of key importance as an initiating factor and may be brought about by one or both of two different mechanisms: (1) contractile instabilities within each sarcomere (with more than one stable velocity for a given load) under certain loading conditions and (2) non-uniform mechanical properties (strength) of different cells leading to a tendency for inhomogeneous stretch of certain regions of the ventricle wall during parts of the cardiac cycle.

One common denominator of the proposed mechanisms is that they are not readily detected in studies using conventional preparations such as isolated proteins or cells already affected by secondary compensatory changes. What is required, in general, are studies on higher organizational levels using cells, tissues, whole hearts or sarcomere like ensembles that contain mutated proteins but that are unaffected by compensatory changes. Furthermore, for any form of mechanical studies to detect the ensemble effects it will be critical to explore the range of loads experienced during the cardiac cycle. The hypothesis, with slight modifications, may also be relevant in the pathogenesis of skeletal muscle myopathies.

### Conflict of interest statement

The author declares that the research was conducted in the absence of any commercial or financial relationships that could be construed as a potential conflict of interest.
