# Supplementary material for: Hypothesis and theory: mechanical instabilities and non-uniformities in hereditary sarcomere myopathies
Source: Front Physiol. 2014 Sep 15;5:350. doi: 10.3389/fphys.2014.00350 (PMC4163974; doi:10.3389/fphys.2014.00350)
Supplement: Supplementary file 1 [file Presentation1.PDF]

Supporting Information

Hypothesis and theory: Mechanical instabilities and non-uniformities in hereditary sarcomere myopathies

by

Alf Månsson[1]

[1]Department of Chemistry and Biomedical Sciences, Linnaeus University, SE-391 82 Kalmar, Sweden

Running title: Mechanical instabilities in myopathies

Model

A modified version of a recent model (Mansson, 2010) was used. This is based on the following kinetic scheme:

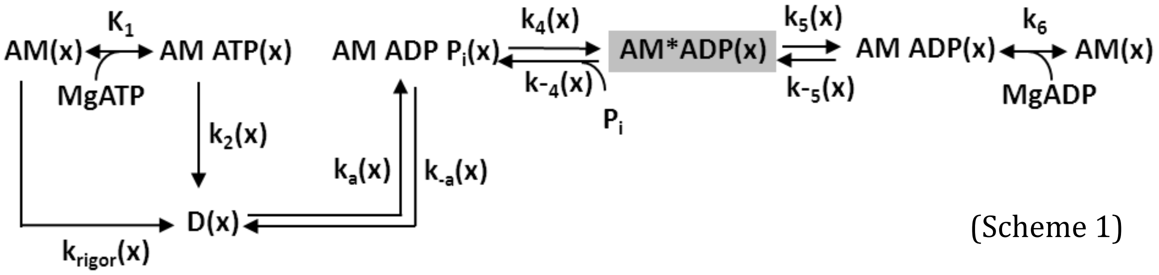

(Scheme 1)

The scheme shows actomyosin (AM) states with either ATP, ADP P_i or ADP (in reality MgATP, MgADP P_i and MgADP) at the myosin active site and a detached state D, lumping together (Persson et al., 2013) myosin-ATP and myosin-ADPP$_i$ states. The argument x indicates that all states (e.g. their structure and free energy; Fig. S1) as well as most rate constants k_2, k_4, *etc.* and equilibrium constants, K_1, K_3, *etc.* vary with the strain parameter x that reports cross-bridge position relative to the nearest actin filament site (Hill, 1974;Eisenberg and Hill, 1978;Persson et al., 2013). However, each different cross-bridge state has its equilibrium position, i.e. its free energy minimum with zero force, at a different x-value (Fig. S1). These equilibrium positions are denoted x_1, x_2 and x_3 for the AM ADP P_i (x), AM*ADP (x) and AM ADP(x) states, respectively. The equilibrium positions of the AM(x) and the AM ATP(x) states are equal to that for the AM ADP(x) state (i.e. x_3) (Persson et al., 2013). With a Hookean cross-bridge elasticity, as assumed here, the free energy of each state (Fig. S1) varies parabolically around the minimal value (Hill, 1974;Eisenberg and Hill, 1978).

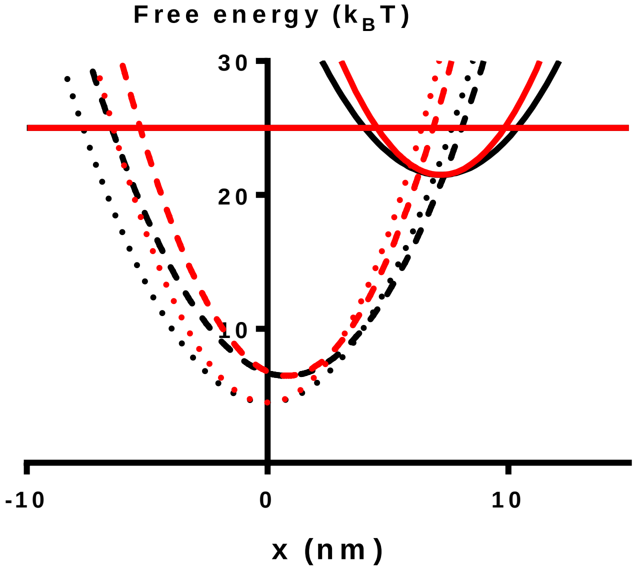

Fig. S1. Free energy diagrams for model states in Scheme 1. Full straight lines: state D, Full curved lines: AMADPP$_i$. Dashed lines: AM*ADP. Dotted lines. AM, AMADP and AMADP states lumped together. Black: standard conditions (Table S1). Red: Standard conditions but cross-bridge stiffness increased by 40 %. See text for further details.

The attachment rate function is given by:

$$k_a(x,v,t) = k_a'(v,t)exp(\Delta G_{d0}/(2k_BT))exp(\frac{-k_c(x-x_1)^2}{2\varepsilon k_BT}) \qquad (S1)$$

where k_c is the cross-bridge stiffness and $k_a'(v,t)$ (in units of s^{-1}) is given below. In some of the simulations (with lengthening during activity) the attachment rate was velocity dependent (see figure legends) as indicated by the argument, v. The parameter ε is a shape parameter related to the width of the Gaussian attachment function. Finally, ΔG_{d0} is the free-energy difference between the D and $AMADPP_i$ states in the absence of cross-bridge strain. Following (Webb et al., 2013), the calcium activation mechanism for muscle contraction was simulated by assuming that the value of the attachment rate function $k_a(x,v,t)$ (Eq. S1) increased with time:

$$k_a'(v,t)=\min(k_{a0}(v,t), k_a(v,\infty)) \qquad (S2)$$

where

$$k_{a0}(v,t)= k_a(v,\infty)\ (fc\frac{Ca(t)^p}{K_{Ca}+Ca(t)^p}+ fm\frac{N}{N_{\frac{1}{2}}+N}) \qquad (S3)$$

Here, the quantity fc is the fraction of maximum activation that can be reached by full calcium binding to the thin filaments whereas fm is the fraction of maximum activation that can be reached by binding of myosin heads in strongly bound states. As previously (Webb et al., 2013), K_{Ca} and p are assumed independent on the number of bound myosin heads. The quantity N is the average number of myosin heads in the AM*D and the AM states. Finally, $N_{1/2}$ is the average number of myosin heads in these states required for half maximal myosin activation. The analysis was simplified by setting fc=1 and fm=0 in Eq. S3.
The velocity dependence in Eqs. S2-3 was only implemented in the simulations in part of main Fig. 3 and in main Fig. 4D. Otherwise, $k_a(v,\infty)= k_{a0}$ for all v. In main Fig. 3, a velocity dependent constant $k_a(v,\infty)$, that gives the rate of attachment at maximum activation, is defined as follows:

$$k_a(v,\infty)= k_{a0} \quad \text{for } v<0 \text{ (shortening)} \qquad (S4a)$$

and

$$k_a(v,\infty)=k_{a0}+1500(1-\exp(-v/500)) \quad \text{for } v>0 \text{ (lengthening)} \qquad (S4b)$$

In these equations, v is in nm/s. In main Fig. 4D, steady-state conditions do not apply. Therefore, the approach in Eqs. S4a-4b to implement velocity dependence could not be used. Instead, increased attachment rate during lengthening was obtained by setting:

$$k_a(v,\infty)=k_{a0}+ 1500\ f_{x>12nm} \qquad (S5)$$

Where $f_{x>12nm}$ is the fraction of attached myosin heads in the AM ADP Pi state for which x> 12 nm. The significance and complications of the different approaches to simulate velocity dependence of the attachment rate is briefly discussed below.
The time variation in Eqs. S2-3 is based on time variation in the intracellular calcium concentration Ca(t) during the onset of a contraction (relaxation is neglected):

$Ca(t)=Ca^{max}(1-exp(-k_{act}t))$ (S6)

where Ca^{max} = 10 µM and k_{act} is set to 100 s^{-1}.

The rate function (in s^{-1}) for reversal of the attachment is given by (as in (Persson et al., 2013)):

$$k_{-a}(x)=min(k_{-a}'(x),20000) \quad \text{(S7)}$$

where

$$k_{-a}'(x)=k_a(x)exp(-\Delta G_{d0}/(2k_BT))exp(\frac{k_c(x-x_1)^2}{2k_BT}) \text{ if } x<11.5 \text{ nm} \quad \text{(S8)}$$

or

$$k_{-a}'(x)=k_a(x)\exp(-\Delta G_{d0}/(k_BT))(k_a(11.5)\exp(\frac{k_c(11.5-x_1)^2}{2k_BT}) \text{ if } x\geq 11.5 \text{ nm} \quad \text{(S9)}$$

The latter function only influences the lengthening part of the force-velocity relation and has no effect on the shortening part or isometric contraction.

The rate function for transition between states has similar shape as previously (Persson et al., 2013):

$$k_4(x)=min(k_4'(x),20000) \quad \text{(S10)}$$

where

$$k_4'(x)=k_{4a}exp(\Delta G_{01}/(k_BT))exp(\frac{-k_c(2(x_1-x_2)x-x_1^2+x_2^2)}{2k_BT}) \quad \text{(S11)}$$

In this equation, k_{4a} is a constant and ΔG_{01} is the difference in minimal free energy between the $AMADPP_i$ and AM*ADP states. The reverse rate function $k_{-4}(x)$ is given by:

$$k_{-4}(x)=k_4(x)exp(-\Delta G_{01}/(k_BT))exp(\frac{k_c(2(x_1-x_2)x-x_1^2+x_2^2)}{2k_BT}) \quad \text{(S12)}$$

and the rate function for transition from the AM*ADP to the AMADP state ($k_5(x)$) is given by:

$$k_5(x)=min(k_5'(x),20000) \quad \text{(S13)}$$

where

$$k_5'(x)=k_{5a}exp(\Delta G_{12}/(k_BT))exp(\frac{-k_c(2(x_2-x_3)x-x_2^2+x_3^2)}{2k_BT}) \quad \text{(S14)}$$

Here, k_{5a} is a constant and ΔG_{12} is the difference in minimum free energy between the AM*ADP and the AM ADP states. This is the strain-dependent transition that is often referred to as a component of the ADP-release with a force-dependent rate (Bell, 1978). The reverse rate function $k_{-5}(x)$ is given by:

$$k_{-5}(x) = k_5(x) exp(-\Delta G_{12}/(k_B T)) exp(\frac{k_c(2(x_2 - x_3)x - x_2^2 + x_3^2)}{2k_B T}) \quad (S15)$$

Finally, an overall detachment rate function (k_{diss}(x, [ATP]) for post-power-stroke cross-bridges is given by:

$$k_{diss}(x, [ATP]) = \frac{k_2(x)k_6[ATP]}{\frac{k_6}{K_1} + (k_2(x) + k_6)[ATP]} = \frac{k_2(x)[ATP]}{\frac{1}{K_1} + \frac{k_2(x)}{k_6}[ATP] + [ATP]} \quad (S16)$$

where k_2(x), k_6 and K_1 are defined in Scheme 1. The strain-dependence of k_2(x) is given by:

$$k_2(x) = k_2(0) \exp\left(\frac{k_c \cdot |x| \cdot x_{crit}}{k_B T}\right) \quad (S17)$$

In accordance with the use of this composite detachment rate function, the states AM ADP(x), AM (x) and AM ATP (x) were lumped together into a new state denoted AM(x) below.
The numerical values of parameters in the above equations are given in Table S1.
In order to obtain steady-state force-velocity data, the following set of equations were solved in the state probabilities *d(x)*, *amdp(x)*, *am*d(x)* and *am(x)* for the states D(x), $AMADPP_i$(x), AM*ADP(x) and the composite state AM(x) at different steady velocities (*v*; negative for shortening):

$$v \frac{\partial d(x)}{\partial x} = k_{diss}(x,[ATP])\, am(x) + k_{-a}(x)\, amdp(x) - k_a(x)\, d(x) \quad (S18)$$

$$v \frac{\partial amdp(x)}{\partial x} = k_a(x)\, d(x) + k_{-4}(x) am^*d(x) - (k_{-a}(x) + k_4(x))\, amdp(x) \quad (S19)$$

$$v \frac{\partial am^*d}{\partial x} = k_4(x)\, amdp(x) + k_{-5}(x) am(x) - (k_{-4}(x) + k_5(x))\, am^*d(x) \quad (S20)$$

$$v \frac{\partial am}{\partial x} = k_5(x)\, am^*d(x) - (k_{-5}(x) + k_{diss}(x,[ATP]))\, am(x) \quad (S21)$$

where, for each x, the sum of the state probabilities is:

$$d(x) + amdp(x) + am^*d(x) + am(x) = 1 \quad (S22)$$

The average force per myosin head (in pN) is obtained as follows if k_c is expressed in pN/nm and x is in nm:

$$\langle F \rangle = \frac{k_c \int_{-\infty}^{\infty} (amdp(x)\cdot(x - x_0) + am^*d(x)\cdot(x - x_1) + am(x)\cdot(x - x_2)) dx}{\int_{-\infty}^{\infty} (d(x) + amdp(x) + am^*d(x) + am(x)) dx} \quad (S23)$$

and the average stiffness due to myosin head attachment is given by:

$$\langle S \rangle = \frac{k_c \int_{-\infty}^{\infty} (amdp(x) + am^*d(x) + am(x)) dx}{\int_{-\infty}^{\infty} (d(x) + amdp(x) + am^*d(x) + am(x)) dx} \quad (S24)$$

For obtaining time courses, the following differential equations were solved repeatedly over very short time intervals (generally 0.00002 s) for each of 800 discrete and equidistant x-

values in the range [-20, 20 nm] before imposing a length change to adjust the cross-bridge strain to match an externally imposed load:

$$\frac{dd(x,t)}{dt} = k_{diss}(x,[ATP])\, am(x,t) + k_{-a}(x)\, amdp(x,t) - k_a(x,t)\, d(x,t) \qquad \text{(S25)}$$

$$\frac{damdp(x,t)}{dt} = k_a(x,t)\, d(x,t) + k_{-4}(x) am^*d(x,t) - (k_{-a}(x)+k_4(x))\, amdp(x,t) \qquad \text{(S26)}$$

$$\frac{dam^*d}{dt} = k_4(x)\, amdp(x,t) + k_{-5}(x) am(x,t) - (k_{-4}(x)+k_5(x))\, am^*d(x,t) \qquad \text{(S27)}$$

$$\frac{dam}{dt} = k_5(x)\, am^*d(x,t) - (k_{-5}(x)+k_{diss}(x,[ATP]))\, am(x,t) \qquad \text{(S28)}$$

Here, $\frac{dd(x,t)}{dt}$, $\frac{damdp(x,t)}{dt}$ etc. are the total derivatives, e.g.:

$$\frac{dd(x,t)}{dt} = \frac{\partial d(x,t)}{\partial x}\frac{dx}{dt} + \frac{\partial d(x,t)}{\partial t}, \text{ etc} \qquad \text{(S29)}$$

The strain adjustment was achieved by shifting the cross-bridge strain by:

$$\Delta x = (F_{set} - \langle F \rangle)/\langle S \rangle \qquad \text{(S30)}$$

before going on to the next time step. Here, F_{set} is the external load (per myosin head) that was kept constant in the simulations.

The steady-state method (Eqs. S18-22) was implemented using the software Simnon (SSPA systems, Gothenburg, Sweden) whereas the time varying method (Eqs. S25-29) was implemented in Matlab (Mathworks, Natick, MA). In both cases a Runge-Kutta algorithm of order 4/5 was used to solve ordinary differential equations. In the time varying method, the force-velocity relationship was obtained by linear fits to simulated length changes vs. time upon imposition of different constant loads (simulating load-clamp recording in muscle) after steady tension had been reached during an otherwise isometric tetanus.

In order to simulate the effect of different contractile properties between cells or between cell segments the simplest possible approach was taken, assuming two segments with different contractile properties acting in series.

This was simulated by changing the parameters in Table S1 for one of the cells as indicated in the respective figure legends (main Fig. 4). The differential equations S25-29 were solved in parallel for the two cells as described above. The update in strain between time steps was achieved as above (Eq. S30) for each cell if the cells were allowed to change length against a constant load. In contrast, in overall isometric contraction, the strain of simulated wild-type (Δx_w) or mutated (Δx_m) cell was updated after each time step as:

$$\Delta x_w = (\langle F_w \rangle - \langle F_m \rangle)/(\langle S_w \rangle + \langle S_m \rangle) \qquad \text{(S31)}$$

$$\Delta x_m = -\Delta x_w \qquad \text{(S32)}$$

For the purpose of the present simulations, parameter values (Table S1) were assumed similar to those for fast rabbit skeletal muscle myosin at 30 ^oC for ATP turnover kinetics and other actomyosin cross-bridge properties, e.g. stiffness. The reason is that these values ((Persson et al., 2013) for references to original studies) have been determined in greater

detail than corresponding data for human heart or skeletal muscle that are, otherwise, in major focus here.

Table S1. Parameter values[a] for fast rabbit skeletal muscle used here

**Parameter**	**Numerical value**	**Source**
k_c	2.8 pN/nm	(Kaya and Higuchi, 2010;Persson et al., 2013)
x_1 (AMADPP$_i$)	7. 20 nm	(Tyska et al., 1999;Kaya and Higuchi, 2010)
x_2 (AM*ADP)	0.8 nm	(Capitanio et al., 2006)
x_3 (AM*ADP)	0 nm	NA
ΔG_{d1}(D- AMADPP$_i$)	3.5 k_BT	(Eisenberg et al., 1980;Pate and Cooke, 1989;Mansson, 2010)
ΔG_{12}(AMADPP$_i$ – AM*ADP)	15 k_BT	(Pate and Cooke, 1989;Howard, 2001)
ΔG_{23} (AM*ADP-AM)	2 k_BT	(Albet-Torres et al., 2009;Mansson, 2010)
ΔG_{ATP}	13.1 + ln ([MgATP]/([MgADP][Pi]) k_BT	(Pate and Cooke, 1989)
ε	1.5	Fitted[c]
k_{a0}	80 s^{-1} [d]	Fitted[c]
k_{4a}	6000 s^{-1}	(Linari et al., 2009)
k_{5a}	2000 s^{-1}	Fitted[c]
x_{crit}	0.6 nm	(Capitanio et al., 2012;Persson et al., 2013)
k_6	5000 s^{-1}	(Nyitrai et al., 2006)
k_{-6}	14 290 mM^{-1} s^{-1}	(Nyitrai et al., 2006)
[MgADP]	0.01 mM	(Pate and Cooke, 1989)
[Pi]	0.5 mM	(Debold et al., 2011)
K_1	1.7 mM^{-1}	(Nyitrai et al., 2006)
$k_2(0)$	1530 s^{-1}	(Nyitrai et al., 2006)
k_{act}	100 s^{-1}	Empiric
Ca^{max}	10 µM	(Webb et al., 2013)
f_c	1	(Webb et al., 2013)
K_{Ca}	2 µM	(Webb et al., 2013)
f_m	0	simplified
P	NA (f_m=0)	
$N_{1/2}$ (% of available heads)	NA (f_m=0)	

NA: Not applicable

[a] Motivated in Persson et al(Persson et al., 2013) but the minimum free energy curve of AM*ADP state modified in accordance with Kaya and Higuchi(Kaya and Higuchi, 2010)

[c]Fit of force-velocity relationship

[d]Attachment rate function chosen to fit maximum power output.

[e]From the assumption of a diffusion limited rate constant > 10 000 mM^{-1} s^{-1} (14 000 mM^{-1} s^{-1}).

Table S2. Effect of small (20-40 %) increase or decrease in isolated parameter values on maximum velocity (V_0), maximum isometric force (F_0) and on the presence of instability in the force-velocity relationship

**Parameter**	**Num. value**	**Up approx 20 % (F_0, V_0, instability?)**[a]			**Down 20 % (F_0, V_0, instability?)**[a]		
		P0	V0	Instability	P0	V0	Instability
k_c	2.8 pN/nm	0.98	0.97	Yes, infinite slope BUT **large effect for 40 % increase with small effect on P0 and V0** (main Fig. 3)	0.99	1.014	No
x_1 (AMADPP$_i$)	7. 20 nm	0.84	1.06	Large effect (strongly positive dV/dP)	1.04	0.86	No
x_2 (AM*ADP)	0.8 nm	1.32	0.95	No	0.72	1.04	No
x_3 (AM*ADP)	0 nm	-	-	-	-	-	-
ΔG_{d1}(D-AMADPP$_i$)	3.5 k_BT	1.18	1	No	0.83	1	No
ΔG_{12} (AMADPP$_i$ –AM*ADP)	15 k_BT	1.22	1.06	No	0.80	0.91	No
ΔG_{23} (AM*ADP-AM)	2 k_BT	0.91	1.04	No	1.10	0.94	No
ε	1.5	1.25	0.99	No	0.78	1.01	No (but close to infinite slope)
k_{a0}	80 s^{-1} [d]	1.05	1	No	0.94	0.99	No
k_{4a}	6000 s^{-1}	1	1	No	1	0.99	No
k_{5a}	2000 s^{-1}	0.97	1.01	No	1.04	0.98	No
x_{crit}	0.6 nm	0.99	1.07	No	1.02	0.91	No
k_6	6000 s^{-1}	0.99	1.07	No	1.02	0.90	No

[a] F_0 and V_0 after change in parameter value given as fractions of values with standard parameter values

Supporting Results

The basic model for the function of a large ensemble of actomyosin motors in muscle was developed from recent, extensively validated, models of similar type (Albet-Torres et al., 2009;Mansson, 2010;Persson et al., 2013). It was therefore not unexpected that the steady-state force velocity (F-V) relationship of a skeletal muscle fiber is well simulated (Fig. S2) using parameter values where most were derived, at least approximately, from the literature (Table S1). Furthermore, as demonstrated for previous versions of the model, typical effects of the drug amrinone (reduced maximum velocity, increased isometric force and changes in shape of the force-velocity relationship) are reproduced (Fig. S2).

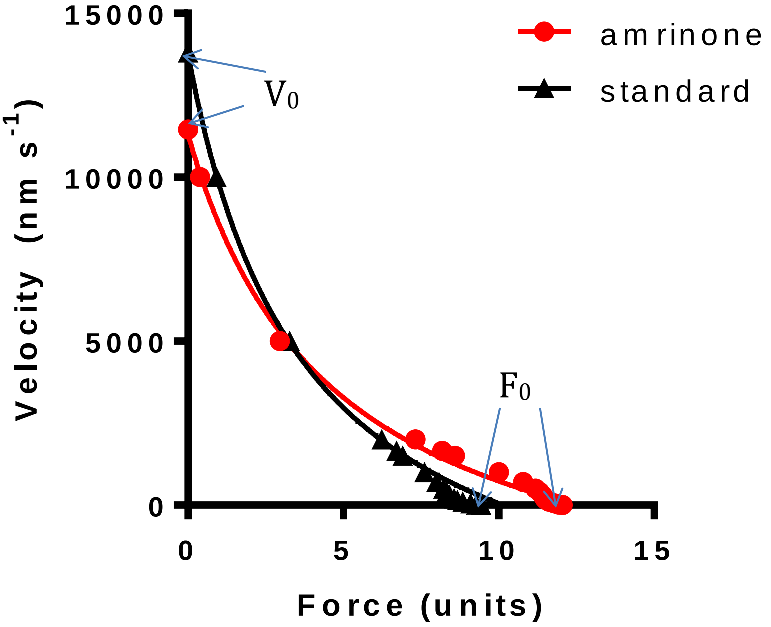

Fig. S2. Simulated force-velocity data. Black symbols: data simulated using parameter values in Table S1 and Eqs. S1-17. Red symbols: Same parameter values as for black symbols except decrease in ΔG_{23} to 0.8 k_BT (see (Albet-Torres et al., 2009;Mansson, 2010;Persson et al., 2013)). Black and red lines represent fits of Hill´s equation (Hill, 1938) to simulated data with parameter a/F_0^* equal to 0.356 (standard) and 0.369 (amrinone), respectively. Arrows indicate maximum velocity of shortening V_0 and maximum isometric force, F_0. The parameter F_0^* represents the intercept of the Hill equation on the abscissa.

Following these initial tests we investigated if any changes in parameter values would lead to an anomalous force-velocity relationship (cf. main Fig. 3) with a region of positive or infinite dV/dF and more than one stable velocity for a given force-level (Julicher and Prost, 1995;Vilfan et al., 1999;Mansson, 2010).

This analysis (Table S2) suggests that moderate (± 20 %) isolated changes in only two of all parameter values tested would lead to instabilities in the force-velocity relationship. These parameters are the cross-bridge stiffness and the strain parameter, x_1, in the pre-power-stroke $AMADPP_i$-state. Interestingly, both these parameter changes operate by a similar mechanism. Thus, both in the case with increased cross-bridge stiffness (see Fig. S1) and increase in x_1 the cross-bridge has to overcome an increased free-energy barrier to enter from the pre-power-stroke state (AMADPPi) into the main force-generating state (AM*ADP). This energy barrier is lowered by the reduced cross-bridge strain during shortening which explains why force can be higher during slow shortening (red, main Fig. 3) than in isometric

contraction. The curves in the main Fig. 3 and Fig. S1 correspond to 40 % increase in cross-bridge stiffness.
The effect of increased stiffness is of particular interest because a mutation-induced change of this type has actually been associated with HCM (Seebohm et al., 2009). Furthermore, both the increase in cross-bridge stiffness and the increased value of x_1 are both associated with only small changes in maximum isometric force and shortening velocity as often seen with HCM-causing mutations in the absence of secondary compensatory changes.

The above model, assuming that all actomyosin cross-bridges have identical kinetic and mechanical properties, was extended in order to incorporate capabilities to elucidate effects of non-uniform contractile properties between cardiomyocytes (~100 µm long) in series or along skeletal muscle fibers (several cm long). Here we approximated this complex arrangement by assuming that two sarcomeres with different contractile properties act in series.
Simulated tension and length changes of two such sarcomeres, with assumed instantaneous activation, are illustrated in Fig. S3. However, in this case the cross-bridge properties are assumed identical for the two sarcomeres, i.e. the two sarcomeres have the same force-velocity relationship (given by black symbols in Fig. S2 and main Fig. 3) including the same isometric tension. First the sarcomeres develop isometric force up to a predetermined level after which the tension was adjusted to the external load (assumed clamped to a constant value), resulting in shortening. As expected, tension develops with identical time course for the two sarcomeres and the length of the two simulated sarcomeres is constant until they are allowed to shorten against an external load. Then each sarcomere shortens at an average velocity that is consistent with the force-velocity relationship in Fig. S2. The superimposed oscillations on the shortening length records are in accordance with previous theoretical and experimental results (Duke, 1999;Edman and Curtin, 2001). However, importantly, these oscillations may be modified by small changes in model characteristics and, in a real muscle the effect is likely to be blunted by non-uniformities between different muscle segments (Duke, 1999). It is also shown in Fig. S3 that the behavior of the two sarcomeres in series is very similar to that of one cell simulated using the same parameter set.

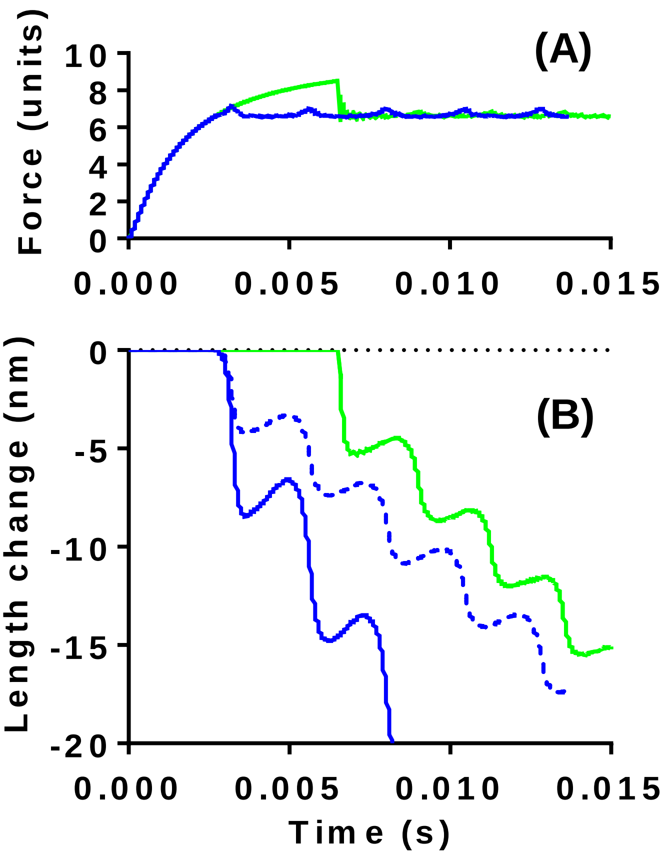

Fig. S3. Simulation of force and length traces for load-clamp experiments were force first increases isometrically, followed by clamping to pre-set level. A. Simulated tension traces. Blue: tension of one cell acting in series with another that behaves identically. Green: other simulation where tension increases nearly to isometric plateau value (9.44 units) before being reduced to a pre-set force clamp level. Small tension ripples were reduced by shortened time steps in simulations. **B.** Length changes. Blue dashed line: length change of one cell in response to force clamp starting after ~3 ms. Blue full line: sum of length change of two identical cells in series. Green: length change for one cell in other simulation (A) with force clamp starting after ~9 ms. Steady-state force-velocity curve of simulated cells according to black filled line in main Fig. 3. Reduced simulation time step had negligible effect on the oscillatory length response. It primarily affected the ripples in the clamped tension. Data simulated using Eqs. S1-17; S23-32 and parameter values in Table S1.

Supporting Discussion

Models of the present type have appreciable potential to help elucidating details of how myopathy-causing mutations affect muscle function prior to development of actual disease. The models thus allow extrapolation to large ensemble of structural and kinetic information deduced from studies of isolated proteins, including single molecule studies. As demonstrated here, it is also feasible to incorporate calcium-regulation and elucidate effects of non-uniformities and other phenomena that operate on a level of hierarchical organization higher than that of the sarcomere. However, in order to take full advantage of the approach it is important to further characterize a number of still poorly understood physiological properties

of heart and skeletal muscle (Sweeney and Houdusse, 2010;Batters et al., 2014) (see also below)

Because more complete data sets exist for fast rabbit skeletal muscle the behavior of such a muscle was simulated here in order to demonstrate principles. However, also in this case, there are uncertainties as to whether contractile activation only increases the number of available sites on the thin filaments (steric blocking model) (Cecchi et al., 1981) or if also the attachment rate constant is increased (Webb et al., 2013). For simplicity only the latter mechanism is assumed to operate which, however, has the effect that the shape of the force-velocity relationship is changed with activation level. Another problem is that it has been found difficult to simulate the force-velocity relationship assuming the same rate of cross-bridge attachment during the onset of an isometric contraction as during shortening with intermediate velocity or fast lengthening (Piazzesi et al., 1992;Edman et al., 1997). Different ideas of how to account for this complexity have been considered (Piazzesi et al., 1992;Mansson, 1994;Piazzesi and Lombardi, 1995;Edman et al., 1997;Huxley and Tideswell, 1997;Brunello et al., 2007;Mansson, 2010;Caremani et al., 2013;Nocella et al., 2013) (see further below). Here it was simply assumed that the rate of cross-bridge attachment is as high during the onset of isometric contraction as in shortening. In most simulations it was assumed that it is similar in lengthening as during shortening and isometric contraction but in the simulation in main Fig. 4D it was assumed that the attachment rate increases with velocity of lengthening. For practical reasons, this was not implemented in the same way as for the steady-state case but, again, this is of no concern considering that the goal of the simulations is to demonstrate possible principles of disease mechanisms rather than giving detailed quantitative predictions. The possibility was not considered that the lengthening response is altered by myopathy mutations.

Supporting References

Albet-Torres, N., Bloemink, M.J., Barman, T., Candau, R., Frölander, K., Geeves, M.A., Golker, K., Herrmann, C., Lionne, C., Piperio, C., Schmitz, S., Veigel, C., and Månsson, A. (2009). Drug effect unveils inter-head cooperativity and strain-dependent ADP release in fast skeletal actomyosin. *Journal of Biological Chemistry* 284, 22926–22937.

Batters, C., Veigel, C., Homsher, E., and Sellers, J.R. (2014). To understand muscle you must take it apart. *Front Physiol* 5, 90. doi: 10.3389/fphys.2014.00090.

Bell, G.I. (1978). Models for the specific adhesion of cells to cells. *Science* 200, 618-627.

Brunello, E., Reconditi, M., Elangovan, R., Linari, M., Sun, Y.B., Narayanan, T., Panine, P., Piazzesi, G., Irving, M., and Lombardi, V. (2007). Skeletal muscle resists stretch by rapid binding of the second motor domain of myosin to actin. *Proc Natl Acad Sci U S A* 104, 20114-20119. doi: 0707626104 [pii]10.1073/pnas.0707626104.

Capitanio, M., Canepari, M., Cacciafesta, P., Lombardi, V., Cicchi, R., Maffei, M., Pavone, F.S., and Bottinelli, R. (2006). Two independent mechanical events in the interaction cycle of skeletal muscle myosin with actin. *Proceedings of the National Academy of Sciences of the United States of America* 103, 87-92.

Capitanio, M., Canepari, M., Maffei, M., Beneventi, D., Monico, C., Vanzi, F., Bottinelli, R., and Pavone, F.S. (2012). Ultrafast force-clamp spectroscopy of single molecules reveals load dependence of myosin working stroke. *Nature methods* 9, 1013-1019. doi: 10.1038/nmeth.2152.

Caremani, M., Melli, L., Dolfi, M., Lombardi, V., and Linari, M. (2013). The working stroke of the myosin II motor in muscle is not tightly coupled to release of orthophosphate from its active site. *The Journal of physiology* 591, 5187-5205. doi: 10.1113/jphysiol.2013.257410.

Cecchi, G., Colomo, F., and Lombardi, V. (1981). Force-velocity relation in deuterium oxide-treated frog single muscle fibres during the rise of tension in an isometric tetanus. *J Physiol (Lond)* 317, 207-221.
Debold, E.P., Turner, M.A., Stout, J.C., and Walcott, S. (2011). Phosphate enhances myosin-powered actin filament velocity under acidic conditions in a motility assay. *American Journal of Physiology: Regulatory, Integrative and Comparative Physiology* 300, R1401-1408. doi: 10.1152/ajpregu.00772.2010.
Duke, T.A. (1999). Molecular model of muscle contraction. *Proc Natl Acad Sci U S A* 96, 2770-2775.
Edman, K.A.P., and Curtin, N.A. (2001). Synchronous oscillations of length and stiffness during loaded shortening of frog muscle fibres. *J Physiol* 534, 553-563.
Edman, K.A.P., Mansson, A., and Caputo, C. (1997). The biphasic force-velocity relationship in frog muscle fibres and its evaluation in terms of cross-bridge function. *J Physiol (Lond)* 503, 141-156.
Eisenberg, E., and Hill, T.L. (1978). A cross-bridge model of muscle contraction. *Prog Biophys Mol Biol* 33, 55-82.
Eisenberg, E., Hill, T.L., and Chen, Y. (1980). Cross-bridge model of muscle contraction. Quantitative analysis. *Biophys J* 29, 195-227.
Hill, A.V. (1938). The heat of shortening and the dynamic constants of muscle. *Proceedings of the Royal Society B 136–195* 126, 136-195.
Hill, T.L. (1974). Theoretical formalism for the sliding filament model of contraction of striated muscle. Part I. *Prog Biophys Mol Biol* 28, 267-340.
Howard, J. (2001). *Mechanics of motor proteins and the cytoskeleton.* Sunderland, MA: Sinauer Associates Inc.
Huxley, A.F., and Tideswell, S. (1997). Rapid regeneration of power stroke in contracting muscle by attachment of second myosin head. *J Muscle Res Cell Motil* 18, 111-114.
Julicher, F., and Prost, J. (1995). Cooperative molecular motors. *Physical review letters* 75, 2618-2621.
Kaya, M., and Higuchi, H. (2010). Nonlinear elasticity and an 8-nm working stroke of single myosin molecules in myofilaments. *Science* 329, 686-689. doi: 10.1126/science.1191484.
Linari, M., Piazzesi, G., and Lombardi, V. (2009). The effect of myofilament compliance on kinetics of force generation by myosin motors in muscle. *Biophysical journal* 96, 583-592. doi: 10.1016/j.bpj.2008.09.026.
Mansson, A. (1994). The tension response to stretch of intact skeletal muscle fibres of the frog at varied tonicity of the extracellular medium. *Journal of muscle research and cell motility* 15, 145-157.
Mansson, A. (2010). Actomyosin-ADP states, inter-head cooperativity and the force-velocity relation of skeletal muscle. *Biophysical Journal* 98, 1237-1246.
Nocella, M., Bagni, M.A., Cecchi, G., and Colombini, B. (2013). Mechanism of force enhancement during stretching of skeletal muscle fibres investigated by high time-resolved stiffness measurements. *Journal of muscle research and cell motility* 34, 71-81. doi: 10.1007/s10974-012-9335-4.
Nyitrai, M., Rossi, R., Adamek, N., Pellegrino, M.A., Bottinelli, R., and Geeves, M.A. (2006). What limits the velocity of fast-skeletal muscle contraction in mammals? *Journal of Molecular Biology* 355, 432-442.
Pate, E., and Cooke, R. (1989). A model of crossbridge action: the effects of ATP, ADP and Pi. *J Muscle Res Cell Motil* 10, 181-196.

Persson, M., Bengtsson, E., Ten Siethoff, L., and Mansson, A. (2013). Nonlinear cross-bridge elasticity and post-power-stroke events in fast skeletal muscle actomyosin. *Biophysical journal* 105, 1871-1881. doi: 10.1016/j.bpj.2013.08.044.

Piazzesi, G., Francini, F., Linari, M., and Lombardi, V. (1992). Tension transients during steady lengthening of tetanized muscle fibres of the frog. *J Physiol (Lond)* 445, 659-711.

Piazzesi, G., and Lombardi, V. (1995). A cross-bridge model that is able to explain mechanical and energetic properties of shortening muscle. *Biophys J* 68, 1966-1979.

Seebohm, B., Matinmehr, F., Kohler, J., Francino, A., Navarro-Lopez, F., Perrot, A., Ozcelik, C., Mckenna, W.J., Brenner, B., and Kraft, T. (2009). Cardiomyopathy mutations reveal variable region of myosin converter as major element of cross-bridge compliance. *Biophysical journal* 97, 806-824. doi: 10.1016/j.bpj.2009.05.023.

Sweeney, H.L., and Houdusse, A. (2010). Structural and functional insights into the Myosin motor mechanism. *Annual review of biophysics* 39, 539-557. doi: 10.1146/annurev.biophys.050708.133751.

Tyska, M.J., Dupuis, D.E., Guilford, W.H., Patlak, J.B., Waller, G.S., Trybus, K.M., Warshaw, D.M., and Lowey, S. (1999). Two heads of myosin are better than one for generating force and motion. *Proceedings of the National Academy of Sciences of the United States of America* 96, 4402-4407.

Webb, M., Jackson Del, R., Jr., Stewart, T.J., Dugan, S.P., Carter, M.S., Cremo, C.R., and Baker, J.E. (2013). The myosin duty ratio tunes the calcium sensitivity and cooperative activation of the thin filament. *Biochemistry* 52, 6437-6444. doi: 10.1021/bi400262h.

Vilfan, A., Frey, E., and Schwabl, F. (1999). Force-velocity relations of a two-state crossbridge model for molecular motors. *Europhysics Letters* 45, 283-289. doi: DOI 10.1209/epl/i1999-00160-3.
